# Treatment of prescription opioid disorders in Canada: looking at the ‘other epidemic’?

**DOI:** 10.1186/s13011-016-0055-4

**Published:** 2016-03-08

**Authors:** Benedikt Fischer, Paul Kurdyak, Elliot Goldner, Mark Tyndall, Jürgen Rehm

**Affiliations:** Social and Epidemiological Research Department, Centre for Addiction & Mental Health (CAMH), Toronto, ON M5S 2S1 Canada; Department of Psychiatry, University of Toronto, Toronto, ON M5T 1R8 Canada; Institute of Medical Science, Faculty of Medicine, University of Toronto, Toronto, ON M5T 1R8 Canada; Centre for Applied Research in Mental Health & Addiction (CARMHA), Simon Fraser University, Vancouver, V6B 5K3 Canada; Mental Health and Addictions Program, Institute for Clinical Evaluative Science (ICES), Toronto, ON M4N 3M5 Canada; B.C. Centre for Disease Control (BCCDC), Vancouver, BC V5Z 4R4 Canada; Department of Medicine, University of British Columbia, Vancouver, BC V5Z 1M9 Canada; Dalla Lana School of Public Health, University of Toronto, Toronto, ON M5T 3M7 Canada

**Keywords:** Prescription opioids, Disorder, Treatment, Opioid maintenance treatment, Evidence-based care

## Abstract

The magnitude and consequences of prescription opioid (PO) misuse and harms (including rising demand for PO disorder treatment) in Canada have been well-documented. Despite a limited evidence-base for PO dependence treatment, opioid maintenance therapy (OMT) - mostly by means of methadone maintenance treatment (MMT) - has become the de facto first-line treatment for PO-disorders. For example in the most populous province of Ontario, some 50,000 patients - large proportions of them young adults - are enrolled in MMT, resulting in a MMT-rate that is 3–4 times higher than that of the United States. MMT in Ontario has widely proliferated towards a quasi-treatment industry within a system context of the public fee-payer offering generous incentives for community-based MMT providers. Contrary to the proliferation of MMT, there has been no commensurate increase in availability of alternative (e.g., detox, tapering, behavioral), and less intrusive and/or costly, treatments which may provide therapeutic benefits at least for sub-sets of PO-dependent patients. Given the extensive PO-dependence burden combined with its distinct socio-demographic and clinical profile (e.g., involving many young people, less intensive or risky opioid use), an evidence-based ‘stepped-care’ model for PO dependence treatment ought to be developed in Canada where MMT constitutes one, but likely a last resort or option, for treatment. Other, less intrusive treatment options as well as the best mix of treatment options should be systematically investigated and implemented. This case study has relevance and implications for evidence-based treatment also for the increasing number of other jurisdictions where PO misuse and disorders have been rising.

By now, the detrimental extent and consequences of the ‘epidemic’ of prescription opioid (PO) misuse and harms in North America are well documented. Despite recent ‘plateauing’ effects, some 4.2 % of adults report non-medical PO use (NMPOU) and some 17,000 PO-related poisoning fatalities occur annually in the United States [1–4]. In Canada, NMPOU prevalence continues to be second only to cannabis among adults and adolescents when compared to illicit drug use. While PO-related poisoning deaths have shifted somewhat between different PO formulations (e.g., oxycodone to hydromorphone or fentanyl) their overall numbers continue to climb and are proportionally similar to those recorded in the US [5–7]. Ample evidence has also shown that the high levels of PO dispensing are the principal driver of the corresponding levels of PO-related harms in North America [1, 8, 9].

A related but commonly overlooked phenomenon concerns the realities of treatment for PO-related disorders. A recent report in the Lancet (2012) concluded that “research into the treatment for [PO] addiction has been chronically neglected. As a result, the evidence base that informs best practice is thin […] The ‘standard treatment’ for [PO] dependence is evolving, and [there is no] single current standard at this time” [10]. Yet, current Canadian treatment system realities seem to suggest the opposite. In Ontario, Canada’s most populous province (~13.6 million pop.), the number of individuals enrolled in methadone maintenance treatment (MMT) has skyrocketed to just under 50,000 in 2014 (from a mere 3000 in 1996, and 29,000 in 2010), with the vast majority of recent enrollments presumed to be PO-related. This situation is in comparison to the United States (US), where there is a similar prevalence of opioid misuse, with opioid maintenance treatment (OMT) being the mainstay similar in design and delivery modes to Canada [11–13]. In the US, however, the total number of OMT clients has increased from some 230,000 (2003) to 340,000 in 2011 - an increase of about 50 % yet making for an OMT enrollment rate that is only about 25–30 % that of Ontario [14]. Importantly, while the number of MMT enrolments in Ontario continues to rise, 29 % of MMT patients (in 2014) were <30 years - and up to 1 in 5 were <25 years in some health regions. MMT patients in this age range likely had relatively short or only tangential involvement with PO misuse prior to treatment, yet likely will be involved in MMT for long periods of time given the lifelong nature of this treatment for many patients [15, 16]. Further, PO disorder patients are commonly more socio-economically integrated and feature lower clinical risk profiles (e.g., less severe addiction severity, less injecting and/or co-morbidities) than the profiles of (largely heroin using) patients that entered OMT pre-2000. Notably, the application of the new DSM-V criteria for PO-disorders is expected to further considerably increase the number of people initiated on OMT [17].

The above data reflect that OMT - mostly with methadone but some suboxone-based in exceptional cases - has proliferated as the de facto first-line treatment for PO-related disorders in Ontario. This is despite the fact that OMT is designed as long-term - in many cases for life - pharmacotherapy for most patients [18]. The predominant reliance on OMT for PO-disorder treatment is mainly based by research evidence from long-term heroin users, even though substantial, clinically relevant differences between heroin and PO users are documented [19–23]. Furthermore, this practice has evolved largely in the absence of an evidence-based stepped-treatment model for PO-disorders, even though evidence exists for benefits of treatment options less intrusive (and potentially less costly) than MMT. For example, several studies - including youth/adolescent patient samples - have shown good select effectiveness involving (buprenorphine-naloxone or naltrexone) medication-supported taper treatments for PO-dependence (with up to 50 % of good treatment outcomes at 3- to 6-months follow-up points) from which many patients would likely benefit [24–27]. Behavioral treatments have both shown some effectiveness as singular treatment interventions as well as in combination with medication-supported detoxification for opioid disorders yet remain under-explored [28–31]. Notably, there neither are any Canadian research studies systematically examining, nor are treatment guidelines universally integrating these alternative treatment modalities for PO-disorders despite the acute and expanding severity of this problem.

While the pharmaceutical industry’s corporate greed and tactics have been popularly blamed - and legally punished - for the PO abuse epidemic (e.g., [32, 33]), economics within the health care system appear to exert an un-desirable dynamic in the realities of treatment for PO disorders. In addition to standard reimbursement for OMT care within Ontario’s public fee-for-service-based health care system, the province introduced additional financial ‘incentives’ in 2011 to entice more community physicians and pharmacies into MMT delivery [34, 35]. In this context, an extensive proliferation of numerous ‘for-profit’ MMT-only clinics occurred focussing on economies-of-scale - i.e., large patient numbers - yet also featuring treatment quality problems (e.g., compromised patient care, inappropriate take-homes or “carries”, excessive urine testing) [36–38]. While the MMT-focussed incentives have created a proliferation of MMT clinics and patients in Ontario, there has been no commensurate investment in short- or mid-term treatment interventions, for example with abstinence, where possible, as a main goal for potentially suitable patient sub-groups. While these treatment interventions may potentially be more care effort- or management-intensive in the acute treatment phase, they be less costly for the system - yet also provide less income for OMT providers or medications producers - in the long run. To illustrate: The current annual public expenditures - or reimbursement fees - for MMT alone in Ontario are estimated to exceed $250,000,000 [39].

Allow us to be perfectly clear: Our position is not ‘anti’-OMT for PO-disorders. In fact, several of the present authors have actively argued for the expansion of OMT availability in Canada when this was still a highly restricted and scarce treatment for the treatment of opioid disorders not so long ago [15, 40]. We believe however that OMT’s proliferation as the first-line-treatment for PO disorders has been propelled to excess by several of the wrong reasons and that an evidence-based stepped-care model - including non-pharmacotherapy/-maintenance components for initial treatment steps - is urgently required to provide a best and most patient-oriented treatment approaches on a system level. Stepped-care models have been promoted and/or implemented for other areas of mental health or substance abuse (e.g., alcohol or nicotine dependence) treatment [41–44]. In essence, what a stepped care model attempts to do is to align treatment from different options, and treatment intensity, based on key characteristics - e.g., based on comprehensive assessment information - that predict patient need, with an overall goal of employing least intensive but most promising treatment on a case-to-case basis. In the specific context of PO disorders, basic treatment options could, for example, include: brief/cognitive-behavioral interventions; medications-supported (short-term) detoxification/tapering; opioid maintenance treatment. If patients do not respond well to their initially assigned treatment, they would be stepped up to more intensive treatment options.

Unquestionably, there are a large number of individuals suffering from PO disorders in Canada who require treatment. While OMT undoubtedly brings therapeutic benefits to many opioid-dependent people, and is the best available therapuetic choice for a large sub-group of patients with PO disorder it also implies the continued exposure of patients to potential correlated adverse effects (e.g., brain structure changes, depression, mortality) of chronic opioid intake - risks that should be minimized especially with young and non-severely dependent patients [45–50]. Long-term OMT should thus surely be an available treatment option in a continuum-of-care, but primarily for non-responders to less intrusive alternatives where these seem reasonably indicated as a first treatment option. In order to implement a stepped-care model for PO disorder treatment in Canada, comprehensive research needs to be conducted, including both on the socio-clinical characteristics of patients predicting success in the different categories of treatment options, as well as the best mix of varying - including non-maintenance - treatment options in a comprehensive stepped-care approach and system. In this context, we also noted with curiosity that the ‘Executive Committee’ of Canada’s ‘Prescription Drug Strategy’ included a senior representative of Reckitt-Benckiser, the then pharmaceutical company manufacturing a principal OMT product (Suboxone) approved in Canada, and has been formally lobbying the Minister of Health for regulatory practice changes in explicit reference to opioid products from other pharmaceutical opioid manufacturers in this role [51]. This mixes competing interests too closely, and would not be acceptable in other comparable arenas of health care policy (see, for example, [52, 53]).

In summary, we urge policy-makers at relevant levels in Canada to both facilitate the development of an evidence-base - building on existent and facilitate the generation of new data required - for effective non-OMT options towards a comprehensive overall continuum-of-care for PO disorder treatment with OMT as a ‘last resort’; related, we urge the correction of the predominant economic parameters in opioid disorder treatment that seem to have unduly influenced the recent excessive expansions of OMT in Ontario, towards a more and overall public health oriented approach to treatment and care. As the burdensome issue of PO misuse and disorders is increasing in other national system contexts [54, 55], the lessons from Canada may provide useful guidance for the development or shaping of treatment options and systems there as well.

## Abbreviations

MMT: methadone maintenance treatment
NMPOU: non-medical prescription opioid use
OMT: opioid maintenance treatment
PO: prescription opioid
